# Applying the health belief model to understand the usage and experience of self-management techniques in knee osteoarthritis: A mixed-methods observational study

**DOI:** 10.1016/j.ijnsa.2026.100534

**Published:** 2026-04-11

**Authors:** Donya Nemati, Navin Kaushal, NiCole Keith, Niki Munk

**Affiliations:** a College of Nursing, The Ohio State University, Columbus, OH, USA; b Department of Health Sciences, School of Health & Human Sciences, Indiana University- Indianapolis, Indianapolis, IN, USA; c Department of Kinesiology, Indiana University-Bloomington, Bloomington, IN, USA

**Keywords:** Self-administered, Osteoarthritis, Health behavior, Health Knowledge, Attitudes

## Abstract

**Background:**

Self-administered knee-massage (S-KM) has the potential to alleviate symptoms in individuals with knee osteoarthritis (OA). Previous studies have shown inconsistent results, highlighting the need for a theoretical framework to identify effective self-massage practices. This study aimed to identify the behavioral determinants related to S-KM by employing an augmented health belief model alongside the theory of planned behavior.

**Methods:**

A total of 268 participants with knee osteoarthritis completed an online survey assessing perceived susceptibility, severity, barriers, facilitators, task response self-efficacy, and cues to action, along with attitudes and intention. Structural equation modelling was used to evaluate the predictive validity of the proposed model. Thematic analysis was conducted to explore qualitative data regarding participants’ experiences in self-managing knee pain.

**Results:**

The findings revealed that S-KM was significantly predicted by intention (β= 0.21, *p*< .014). Intention was influenced by cues (β= 0.29, *p*< .001), task self-efficacy (β= 0.29, *p*< .001), affective attitudes (β= 0.14, *p*= .011), perceived severity (β = 0.27, *p*< .001), and perceived facilitators (β= 0.22, *p*< .001). Intention mediated the relationship between cues and behavior. Qualitative analysis indicated that many participants used techniques similar to Swedish massage, often due to familiarity from professional massages. Some also reported using massage devices, suggesting a potential shift toward incorporating technology in future self-massage research.

**Conclusions:**

Key predictors of knee massage performance included cues, perceived severity, and task self-efficacy. Interventions should enhance perceptions of OA severity and confidence in self-massage techniques, providing insights for future program development.

**What is already known**

• Self-administered knee massage is potential for symptom relief in knee osteoarthritis but theoretical models guiding interventions are limited.
• Health behavior theories are recommended to inform intervention design for health-promoting behaviors.
• Prior work lacks an integrated framework combining the Health Belief Model with constructs from the Theory of Planned Behavior for Self-administered knee massage.

**What this paper adds**

• The study extends the Health Belief Model by incorporating Theory of Planned Behavior constructs to identify determinants of Self-administered knee massage in individuals with knee osteoarthritis.
• The findings revealed that cues, perceived severity, and task self-efficacy are key predictors of intention to perform Self-administered knee massage, which mediates behavior.
• The results provide a theoretically informed framework to guide the development of future interventions promoting Self-administered knee massage in musculoskeletal conditions.

Alt-text: Unlabelled box dummy alt text

Knee Osteoarthritis (knee OA) is the most common type of arthritis with no recognizable cure (Mahmoudian et al., 2021). Knee OA has been identified as a national public health problem (Araujo et al., 2016; van Doormaal et al., 2020) which leads to around 600,000 knee replacements annually (Cleveland et al., 2013), resulting in expenses of approximately $22 billion each year (Bhatia et al., 2013). Given that knee OA is correlated with age, the prevalence of this disease is expected to increase over the next two decades (Atkins and Eichler, 2013; Flowers et al., 2019; Gustafsson et al., 2020; Wallace et al., 2017). The mobility restrictions, knee pain and stiffness manifest mental health symptoms such as increase in anxiety (Scopaz et al., 2009), decline in quality sleep (Gilbert et al., 2021), and quality of life (Lespasio et al., 2017; Youngcharoen et al., 2020). The large proportion of individuals affected by knee OA necessitates investigation for effectively promoting self-management behavior (Bhatia et al., 2013; Roos and Juhl, 2012; Uritani et al., 2021).

Self-management aims to enhance “the individual’s ability to manage the symptoms, physical treatment, psychological consequences, and lifestyle changes inherent in living with a chronic condition” (Uritani et al., 2021, p. 2) and carries significant importance for individuals with knee OA. For instance, based on the American College of Rheumatology/ Arthritis Foundation guidelines and OA treatment pyramid (Kolasinski et al., 2020; Roos and Juhl, 2012), the primary forms of treatment include non-pharmacological modalities such as education and lifestyle modification which are forms of self-management.

Multiple rehabilitation modalities have been tested for efficacy of symptom management, however, there has been a recent exploration into the use of massage therapy as a self-management approach for relieving knee symptoms. (Atkins and Eichler, 2013; Oka et al., 2020; Tosun et al., 2017). Self-administered knee massage (S-KM) has shown to alleviate pain, stiffness, and improve physical function (Atkins and Eichler, 2013; Munk et al., 2021; Oka et al., 2020; Tosun et al., 2017) while providing as safe, economical approach that empowers individuals to manage their symptoms. This supports adopting S-KM as a co-adjunct to conventional treatments, such as medication and physical therapy (Chan et al., 2015). While more research is needed to fully understand the underlying mechanisms that explain the effectiveness of S-KM current evidence in the literature is not supported by a theoretical framework that establishes the behavioral determinants of performing, adopting, and adhering to S-KMs. These factors are crucial to designing behavior change interventions, including self-management techniques (Goff et al., 2022).

## 1 The process of conceptual model development

We developed a conceptual model for performing S-KM, drawing from both theoretical and empirical literature to identify the behavioral determinants that are the strongest predictors of the S-KM behavior, in addition to account for specific clinical characteristics unique to knee OA. While massage therapy has been gaining popularity as a self-management modality, the literature is limited on investigations of theories or models in this area. To fill this gap, we considered studies that focused on self-management for knee OA to draw from theoretical constructs and develop a conceptual model. Two prominent theories that are frequently employed in self-management literature, consist of the Health Belief Model (HBM) (Rosenstock, 1966, 1974) and the Theory of Planned Behavior (TPB) (Ajzen, 1991). While these theories include some overlapping determinants, relevant constructs to S-KM from the TPB can complement the HBM, which would result in a comprehensive model to advance our theoretical understanding of promoting S-KM and direct research to develop theory-guided interventions.

## 2 Theoretical foundations and linkage to self-management

### 2.1 Health belief model

The Health Belief Model (HBM) is one of the earliest proposed models for understanding and predicting health-related behaviors, particularly engagement in preventive behaviors (Rosenstock, 1966, 1974). HBM suggests that behavior is determined by several cognitive factors, including self-efficacy, perceived threat, cues to action, perceived benefits, and barriers. Cues to action can be from internal (e.g., pain that prompts knee massage) or external sources (e.g., massage roller and healthcare referral). Perceived threat is a combination of perceived susceptibility and perceived severity of negative health outcomes related to illness. Perceived benefits are cognitive beliefs that justify the rewards of performing the behavior. Self-efficacy can be defined as the perceived confidence to perform a particular behavior with sufficient skills (task self-efficacy) and to the extent that it would result in health benefits (response self-efficacy) (Bandura, 1977, 1997). Barriers can be physical or perceived and can include obstacles such as accessibility and lack of skills, fatigue, and time. The Health Belief Model is commonly used to understand various self-management behaviors (Li et al., 2021; Vincenzo et al., 2022).

### 2.2 Theory of planned behavior

The Theory of Planned Behavior (TPB) was developed to predict human social behavior (Ajzen, 1991). TPB posits that intention, defined as commitment, determination, or plan, is a proximal determinant of behavior and is formulated by attitudes, subjective norms, and perceived behavioral control. Attitudes reflect beliefs about a behavior and can be instrumental (related to health impact) or affective (related to enjoyment). Subjective norms represents the extent of support from influential individuals (e.g., physician) or groups. Perceived behavioral control assesses an individual's skills, ability, and capacity to perform the behavior. Antecedents, including demographic variables such as age, gender, socioeconomic status, and location, directly affect these determinants and indirectly affect intention. Attitudes, perceived behavioral control, and subjective norms function as parallel mediators between antecedents and intention in a parallel manner. TPB has been shown to have high predictive validity for self-management behaviors, making it a viable framework to be used in behavior change studies (Jormand et al., 2022; Morowatisharifabad et al., 2020).

### 2.3 Augmented health belief model

**Fig. 1:**
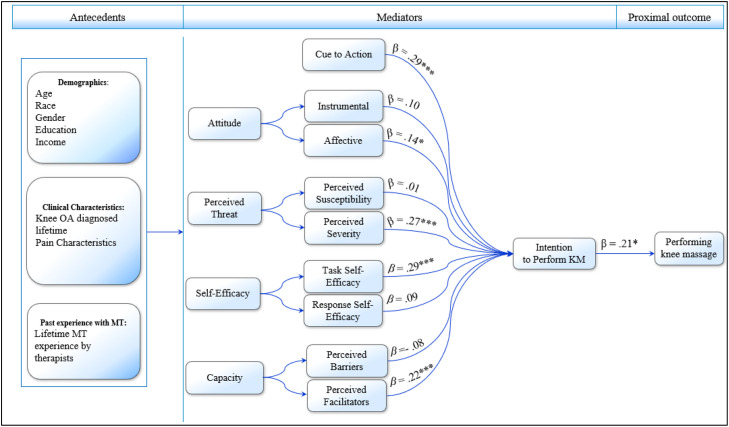
Proposed Conceptual Model. Note: An augmented health belief model for performing self -administered knee massage. The behavior was predicted by intention and intention was predicted by cue to action, affective attitude, perceived severity, task self-efficacy and perceived facilitators. Note. Effect sizes are standardized parameter estimates from a structural equation model. **p < .001, * *p* < .01. β represents standardized coefficients. MT stands for massage therapy, and OA denotes Osteoarthritis.

To propose a conceptual model that incorporates relevant theoretical constructs to perform S-KM the proposed conceptual model advances from the HBM framework with integrating with two constructs of TBP. Specifically, attitude and intention to perform the behavior were supplemented in the HBM model. Ajzen (2020) defines intention as reflection of motivation as its strength is represented by preceding determinants. Hence, intention functions as bridge that manifests beliefs to behavioral enactment. While the HBM includes benefits, attitudes represents a more comprehensive construct as it includes affective and instrumental components, and hence, we replaced benefits with attitudes as we theorize this construct to capture benefits and feeling states of performing behavior. Perceived behavioral control was not transferred as it is quite similar to self-efficacy (Ajzen, 2002), and hence including this construct in the model would likely result in multicollinearity. Given that the nature of self-management approaches is very individual-based behavior and usually performed at home, the construct of subjective norms from TPB, defined as the social acceptance of a behavior, was not relevant and hence, was omitted. Finally, previous work identified the importance of capability, as reflected by barriers and facilitators to perform S-KM (Nemati et al., 2024). We theorize these constructs to be positioned parallel to other HBM determinants and also predict intention. Fig. 1 depicts the augmented model that integrates relevant constructs from HBM and TPB to predict S-KM behavior among individuals with knee OA. The set of intention determinants include attitudes (instrumental and affect), perceived threat (susceptibility and severity), self-efficacy (task and response), barriers, facilitators, and cue to action. These constructs were selected based on demonstrating predictive validity in research pertaining to self-management and/or facilitating health behavior among clinical population groups (Hagger et al., 2019; Keogh et al., 2015; Norozi et al., 2020; Schulman‐Green et al., 2016).

### 2.4 Study purpose

The purpose of this observational study was to test an augmented health belief model to identify significant and strongest determinants for promoting S-KM. The primary objective was to test the predictability of the proposed pathways of the augmented conceptual model. Congruent with the proposed model, we hypothesized S-KM to be directly predicted by intention (H_1_). Intention, in turn would be predicted by cues (H_2_), perceived: severity (H_3_), susceptibility (H_4_), affective attitudes (H_5_), instrumental attitudes (H_6_), task self-efficacy (H_7_), response self-efficacy (H_8_) along with barriers (H_9_), and facilitators (H_10_) (Fig. 1). The secondary objective tested indirect effects. It was hypothesized that intention would mediate between behavior and cues (H_11_), perceived susceptibility (H_12_), and perceived severity (H_13_). The final objective was an exploratory investigation to identify antecedents that carried the strongest impact on determinants and if any predicted behavior via indirect effects. In an exploratory and qualitative approach, we also aimed to identify and gain a better understanding of the execution of S-KM by participants, including techniques they used.

## 3 Methods

### 3.1 Participants

An observational mixed methods study was designed to recruit participants using Cloud Research. Participants were recruited using Cloud Research, which is an American recruitment platform that has >50 million participants who are interested in participating in behavioral research (Verma et al., 2021). Cloud Research is a commonly used platform for recruiting participants for behavioral science studies (Kaushal et al., 2020, 2021) as the users have been shown to be highly representative of the US population (Verma et al., 2021). The eligibility criteria for the proposed study include participants who are adults (at least 18 years of age) and who reported to either having chronic knee pain (>3 months) or physician-diagnosed knee osteoarthritis. To identify eligible individuals, respondents were asked to complete two screening questions which included, i. “Have you ever been diagnosed with Knee Osteoarthritis by a physician?” and ii. “If no, have you been experiencing knee pain for >3 months?”. Eligible participants were then presented with the study consent form and those who agreed to consent were able to access the study survey. The study was approved by the Institutional Review Board at (masked).

### 3.2 Measures

Following the consent form, participants were presented with the definition of S-KM which read, “self-administered knee massage is a self-treatment technique for the personal management of conditions or symptoms that respond to touch, manual, soft tissue manipulation or massage therapy. Any technique you use on your knee such as gliding, tapping, friction strokes, or rhythmically massaging your knee, is considered as a self-administered massage” (D. V. Atkins and D. A. Eichler, 2013). Participants were instructed to keep this definition in mind when completing the survey which included the following measures.

### 3.3 Antecedents

The conceptual definition of antecedents for both Theory of Planned Behavior and Health Belief Model, are essentially identical. Antecedents represent factors, or external variables that determine how theorized determinants in the model may function. For the proposed augmented model, one of the antecedent variables, knee pain history, was operationally defined and measured by the Knee Pain Screening Tool (KNEST), which is a valid and reliable tool (Jinks et al., 2003). In addition, to measure the chronicity of knee pain, a question asks participants to “think back over the last 12 months on how many days have you had knee pain”. Response options include “<7 days,” “1–4 weeks,” “more than one month but less than three months,” and “more than three months”. To record the intensity of knee pain, the Numerical Rating Scale (NRS) was administered to measure pain characteristics (Alghadir et al., 2018; Ornetti et al., 2011), which has demonstrated to be a valid and reliable instrument to measure knee pain (Hawker et al., 2011; Ornetti et al., 2011). The study instructed the participants to assess their knee pain on a scale of 0 to 10 in three different pain domains: current pain, average or typical pain, and the worst pain. The sum of these three ratings was multiplied by 10 to compute the average score. Afterward, the total score was divided into two categories: high (≥50) and low (<50) intensity of pain (Von Korff et al., 1993).

### 3.4 Model constructs

As established in the literature, the best format to evaluate belief, attitude, and opinion is the Likert scale (Waltz et al., 2010), a continuum from strong disagreement to strong agreement. Hence, a 5-point Likert scale consists of a series of statements to that a participant is to indicate a degree of agreement or disagreement using the following response options: strongly disagree=1, disagree = 2, undecided = 3, agree = 4, strongly agree = 5. The higher score in each construct will indicate the more significant perception of the latent variable. Participants were provided with the following instructions at the beginning of the survey, “For each question, please think of performing 10-minute self-administered massage for your knee per day, select the answer that best describes your belief about that statement”. The dosage in the statement was selected based on a clinical trial by Atkin et al., 2013 (Dorothea V Atkins and David A Eichler, 2013).

### 3.5 Instrumental and affective attitudes

Instrumental and affective attitudes measures were adopted from Ajzen’s (1991) attitudes scale (Ajzen, 1991). Affective attitude was defined as the feelings associated with performing S-KM. This construct included items such as, “performing self-administered massage daily for at least 10 min would be…i) beneficial, ii) pleasant iii) valuable”. Instrumental attitudes is defined as the belief about health benefits of performing self-massage. The following items were used to assess the instrumental attitudes construct: “performing self-administered massage daily for at least 10 min would i) reduce pain, ii) improve mobility, iii) improve knee range of motion, iv) reduce knee stiffness, v) would make daily activities easier”. The internal consistency for these measures were α =0.87 and α =0.88, respectively.

### 3.6 Perceived susceptibility and perceived severity

Perceived susceptibility is the likelihood of experiencing worsening outcomes of knee osteoarthritis. The susceptibility scale was adopted from Ang et al. (2008); (Ang, Monahan, et al., 2008) and that included the following four items: i) “The chance that my arthritis will get worse are great”, ii) “due to the condition of my physical health, my arthritis is likely to get worse”, iii) “I worry a lot about my arthritis getting worse”. Perceived severity is operationally defined as “an individual’s perception of the medical or social consequences of having arthritis” (Ang, Shen, et al., 2008) that was tested by items including, “My arthritis keeps me from doing things I want to do” “my arthritis limits my daily activities”, adopted from Ang et al. (2008); (Ang, Monahan, et al., 2008). Perceived susceptibility α = 0.90 and severity α = 0.83 demonstrated to be reliable.

### 3.7 Task self-efficacy and response self-efficacy

Task self-efficacy is defined as the certainty about the ability to perform S-KM. Items for this measure include, “For each of the following statements, please think of performing 10-minute self-administered massage for your knee per day, select the answer that best describes your belief about that statement”, “I am certain that I can apply 10 min of massage/day if I want to”, “I am certain that I can adopt massage in my daily routine”. Response self-efficacy is the certainty of acquiring the health benefits of performing self-massage. This measure was administered with the following items, “I am certain that I can reduce my knee pain by applying self-massage for my knee”, “I am certain that I can improve my mobility by applying self-massage for my knee”. Items were adopted from a scale to measure ‘perceived self‐efficacy in people with arthritis’(Lorig et al., 1989). Cronbach alphas for task (α = 0.90) and response self-efficacy (α = 0.87) were found to be strong.

### 3.8 Perceived barriers and perceived facilitators

Perceived barriers are defined as obstacles and limitations that might hinder performing S-KM for knee osteoarthritis. Perceived barriers was assessed with items that include: i) “Applying self-administered massage for my knee requires a lot of skills which is difficult”, ii) “Applying self-administered massage for my knee can be time-consuming”, iii) “Applying self-administered massage for my knee can be painful”. Perceived facilitators are defined as physical and psychological factors that help to perform S-KM easier. The following items were used to measure this construct: for performing self-administered knee massage regularly, I have “a) sufficient time, b) motivation, c) physical ability (sufficient energy). Perceived barriers and facilitator scales were adopted from Osteoporosis Health Belief Scale (Kim et al., 1991). Internal consistency for perceived barriers and perceived facilitators were found to be α =0.84 and α =0.73, respectively.

### 3.9 Cues to action

Cues to action is defined as any stimulus associated with performing knee massage that could be internal (pain, or stiffness), or external (a health care professional’s referral). Cues can be a direct prompt or reminder from an influential figure, originally operationalized by Rosenstock (1966); (Rosenstock, 1974), such as, “I would perform self-administered massage for my knee if a health professional suggested me to do so”. Additionally, cues can also function as symptomatic triggers, which were assessed by asking participants, “I usually start massaging my knee when my knee joints hurt”, and “I usually start massaging my knee when my knee is stiff”. Cronbach alpha for cues measure was found to be α =0.77.

### 3.10 Intention

Intention is defined as perceived likelihood of performing knee massage daily in the upcoming month that were tested by items such as “I intend to perform self-administered massage daily for 10-min for my knee for the next month”, “It is likely I will perform self-administered massage daily for 10-min for my knee for the next month”, “I expect to perform self-administered massage daily for 10-min for my knee for the next month” which are adopted from Ajzen’s (1991) Intention measure (Ajzen, 1991). Internal consistency for intention was found to be strong (α = 0.89).

### 3.11 Self-Administered massage behavior

The target behavior (self-administered knee massage) is defined based on the literature and as a self-treatment technique for the personal management of conditions or symptoms that respond to touch, manual, soft tissue manipulation or massage therapy. Any technique participants use on their knee such as gliding, tapping, friction strokes, or rhythmically massaging their knee is considered as a self-administered massage [10]. Therefore, the frequency of performing knee massage per week, was calculated by multiplying session by minutes when participants answer the following questions: *Do you perform any knee massage techniques (*e.g.*, rubbing, gliding, tapping, friction strokes, or rhythmically massaging) for your knee symptoms at home? 1. Yes 2. No* followed by “Specifically, over the past 2 weeks, how many times per week, on average, did you perform knee massage at home, and what was the duration?”. The scale framework was adopted from another behavior measure, Godin Leisure-Time Exercise (GLTEQ) (Godin et al., 1986), which is able to capture self-report exercise frequency and duration with high reliability. Participants also were asked, “Would you briefly describe how you perform massage for your knee?” to provide qualitative data for massage performance.

### 3.12 Analysis plan

SPSS and AMOS 24 (Arbuckle, 2014) was used to conduct all analyses. Following reporting of participant descriptives, bivariate correlations among proposed model constructs were performed. Significant correlations were supported to be tested in the proposed conceptual, structural equation model (Fig. 1) which were estimated by using full-information maximum likelihood approach. Latent variables that include self-efficacy, cues, perceived threats, perceived barriers, attitudes and intention were created by using items from their respective scales. The model was controlled by demographic antecedents. Missing data analyses were performed to identify percentage of missing data and if the data is missing at random using Little's MCAR Test. Regression imputation was performed to estimate missing data values if needed (Awang, 2012). Sample size requirements proposed by Wolf et al. (2013) were followed to estimate sample size which revealed for incorporating 4 constructs in the augmented model and an average of 4 to 6 items per construct, with a minimum factor loading of 0.65, we need approximately 250 participants to test the proposed model (Wolf et al., 2013). Model fit were determined by a set of parameters that include Tucker–Lewis Index (TLI), comparative fit index (CFI), Tucker-Lewis Index (TLI), standardized root mean square residual (SRMR) and root mean square error of approximation (RMSEA). Acceptable fit was assessed by CFI and TLI achieving values over 0.90, and values lower than 0.08 and 0.09 for the SRMR and RMSEA respectively (Marsh et al., 2004).

Qualitative data on performing massage were analyzed using thematic analysis which is a suggested approach by Kiger. et al. (2020) for understanding behaviors (Kiger and Varpio, 2020). Specific steps of thematic analysis were conducted including familiarizing with the data, generating initial codes, searching for themes, reviewing themes, defining and naming themes, and the final steps would be interpreting the findings (Kiger and Varpio, 2020).

## 4 Results

### 4.1 Participant demographics and correlations

Table 1 displays the demographic information of the participants (*n* = 269). Among the participants, 48.3% (*n* = 130) were categorized as adults with an average age of 49.21± 9.7, while 51.3% (*n* = 138) were classified as older adults with an average age of 70.91 ± 3.83. The majority of the participants were female (60.9%) and Caucasian (85.5%). Only 42.3% (*n* = 114) of all participants reported having a household income that they considered “enough to make ends meet”.

**Table 1:** Demographic characteristics of participants.

	Younger adults (<65) *n* = 130	Older adults (≥65) *n* = 138	P value (statistical test value)b
**Age, mean (SD)**	49.21 (9.7)	70.91 (3.83)	
**Sex**			
Male	45 (34.6)	59 (42.8)	.210 (χ2 = 1.86)
Female	85 (65.4)	79 (57.2)	
**Race/ Ethnicity**			
Caucasian	103 (79.2)	127 (92.0)	
African American	16 (12.3)	5 (3.6)	
Asian	0 (0.0)	3 (2.2)	.005 (χ2 = 16.74)
Hispanic	3 (2.3)	2 (1.4)	
American Indian or Alaska Native	1 (0.8)	0 (0.0)	
**Household Income**			
Comfortable	45 (34.6)	69 (50.0)	
Just enough to make ends meet	40 (30.8)	37 (26.8)	
Undecided	24 (18.5)	21 (15.2)	.055 (χ2 = 9.27)
Not enough to make ends meet	19 (14.6)	11 (8.0)	
**Job Status**			
Homemaker	17 (13.1)	1 (0.7)	
Retired	30 (23.1)	117 (84.8)	
Social welfare	6 (4.6)	1 (0.7)	
Temporarily unemployed	12 (9.2)	3 (2.2)	>0.001 (χ2 = 107.51)
Full-time employed	51 (39.2)	8 (5.8)	
Part-time employment	14 (10.8)	8 (5.8)	
**Marital Status**			
Never married	26 (20.0)	16 (11.6)	
Married or common law	64 (49.2)	73 (52.9)	.300 (χ2 =3.67)
Separated, divorced, widowed	39 (30.0)	48 (34.8)	
**Education**			
High school or less	20 (15.6)	10 (6.9)	
Some college/associate degree	12 (9.4)	52 (37.9)	
College graduate	65 (50)	48 (34.5)	.381 (χ2 =6.38)
Graduate degree	33 (25)	28 (20.6)	
**Caregiving role**			
Yes, for an older adult	16 (12.3)	11 (8.0)	
Yes, for a child or adult with disability	16 (12.3)	4 (2.9)	.300 (χ2 =13.92)

^a^Values are no (%) unless otherwise indicated. Values may not sum to total because of missing data.

^b^One-way analysis of variance was used for continuous variables, and the χ^2^ test was used for categorical variables.

Table 2 displays the clinical characteristics of the participants in this study. Among knee OA cases, 41.2% (*n* = 112) were bilateral. The average duration of knee diagnosis was 8.8 ± 9.3 years for adults and 14.2 ± 12.4 years for older adults. The total pain score revealed that adults experienced significantly more severe knee pain (64.7 ± 17.8) than older adults (50.0 ± 22.7) (Table 2). As a result, the majority of adults (82.4%) were classified as having high intensity pain. Only 26.3% (*n* = 71) of all participants had a normal BMI, while the rest were classified as overweight up to obesity level III (Table 2). The utilization of therapist-applied massage at any point in their life (yes/no) was 56.5% among adults and 38.7% among older adults, with significant differences between the two groups. Bivariate correlations found intention to correlate with behavior (*r* = 0.40, *p* < .001), and all determinants including cues (*r* = 0.39, *p* < .001), perceived severity (*r* = 0.31, *p* < .001), and task self-efficacy (*r* = 0.60, *p* < .001). Remaining correlations can be found in Table 3.

**Table 2:** Participants clinical profile.

	Younger adults *n* = 130	Older adults *n* = 138	P value (statistical test value)b
**Knee Osteoarthritis History and Profile**			
**Laterality of knee pain**			
Unilateral	80 (61.1)	76 (55.5)	.211 (χ^2^ =0.862)
Bilateral	51 (38.9)	61 (44.45)	
**GP consultation in last year**			
Yes	36 (27.7)	67 (48.9)	<0.001 (χ^2^ =12.67)
No	94 (72.3)	70 (51.1)	
**Days in pain in last 12 months**			
<7 days	11 (8.5)	21 (15.3)	
7 days to 4 weeks	27 (20.8)	14 (10.2)	.054 (χ^2^ =7.622)
1 month to <3 months	20 (15.4)	21 (15.3)	
≥3 months	72 (55.4)	81 (59.1)	
**History of knee pain, mean (SD) [range], y**	8.8 (9.3) [1–40]	14.2 (12.4) [1–50]	<0.001 (*F* = 16.08)
**History of knee OA diagnosis, mean (SD) [range], y**	6.3 (6.9) [1–30]	11.0 (9.1) [1–37]	<0.001 (*F* = 21.67)
**Total VAS pain, mean (SD) [range]**	64.7 (17.8) [13.3–96.6]	50.0 (22.7) [3.3–86.7]	<0.001 (*F* = 34.21)
Current pain	5.3 (2.7) [0–9]	3.45 (2.8) [0–9]	<0.001 (*F* = 30.05)
Average pain	6.1 (2.1) [0–10]	4.6 (2.4) [0–10]	<0.001 (*F* = 28.54)
Worst pain	8.0 (2.6) [1–10]	6.9 (2.4) [0–10]	<0.001 (*F* = 16.88)
**VAS pain intensity category**			
Low intensity	23 (17.6)	61 (45.5)	<0.001 (χ^2^ =23.9)
High intensity	108 (82.4)	73 (54.5)	
**Massage Therapy Utilization**			
Ever in Lifetime (yes)	74 (56.5)	53 (38.7)	.003 (χ^2^ =8.51)
Estimated sessions in Lifetime	12.1 (10.1) [1–30]	13.2 (10.5) [1–35]	.593 (χ^2^ =0.287)
**Prevalence of Obesity**			
**Body Mass Index (kg/m2)**			
Underweight: < 18.5	3 (2.6)	0 (0.0)	
Normal weight: 18.5–24.9	45 (38.5) *	26 (20.0)*	
Overweight: 25.0–29.9	19 (16.2)	34 (26.2)	
Obesity I:30.0–34.9	21 (17.9)	33 (25.4)	<0.001 (χ^2^ = 26.18)
Obesity II:35.0–39.9	3 (2.6) *	18 (13.8)*	
Obesity III:40.0 +	26 (22.2)	19 (14.6)	

^a^Values are no (%) unless otherwise indicated. Values may not sum to total because of missing data.

^b^One-way analysis of variance was used for continuous variables, and the χ^2^ test was used for categorical variables.

*Denotes a subset of age groups categories whose column proportions were significantly different from each other at the 0.05 level.

**Table 3:** Bivariate Correlations between model constructs.

	Construct	1	2	3	4	5	6	7	8	9	10	11
1	Intention		.392**	.537**	.483**	.650**	.601**	.639**	−0.249**	.238**	.305**	.398**
2	Cues			.312**	.267**	.307**	.206**	.264**	.196**	.133*	.223**	.161**
3	Instrumental Attitudes				.460**	.606**	.420**	.405**	−0.061	.229**	.231**	.256**
4	Affective Attitudes					.585**	.531**	.425**	−0.208**	0.067	0.079	.186**
5	Response Self-efficacy						.573**	.517**	−0.308**	.143*	.205**	.314**
6	Task Self-efficacy							.691**	−0.395**	0.103	0.099	.246**
7	Facilitators								−0.355**	0.042	0.016	.200**
8	Barriers									.309**	.321**	−0.022
9	Perceived Susceptibility										.582**	.180**
10	Perceived Severity											.200**
11	Self-administered massage behavior											

Note. The table presents zero-order bivariate and model correlations (values within brackets). Behavior = Performing 10-minute knee massage. ** *p* < .001.

### 4.2 Structural equation model

The SEM model demonstrated strong fit indices (**χ** ^2^ = 1028.38, df = 537, *p*< .001; RMSEA = 0.06 (95% CI = 0.053 to 0.064); TLI = 0.91; CFI = 0.92; SRMR = 0.08). Means, standard deviations and factor loadings can be found in Table 4. Analysis of missing data revealed 12.8% of the data was found to be missing and was missing at random MCAR, *p* > 0.05.

**Table 4:** Mean (M), Standard Deviations (SD), and Factor Loadings of Study Items.

Indicator	Factor Loadings	Means/SD
1. Intention 1	0.83	3.78 (1.16)
2. Intention 2	0.85	3.85 (1.12)
3. Susceptibility 1	0.88	3.56 (1.08)
4. Susceptibility 2	0.89	3.61 (1.03)
5. Susceptibility 3	0.83	3.31 (1.05)
6. Severity 1	0.93	3.44 (1.21)
7. Severity 2	0.89	3.59 (27.40)
8. Instrumental Attitude 1	0.70	3.40 (0.97)
9. Instrumental Attitude 2	0.71	3.40 (0.99)
10. Instrumental Attitude 3	0.69	3.81 (0.91)
11. Instrumental Attitude 4	0.81	3.73 (0.92)
12. Instrumental Attitude 5	0.83	3.68 (0.91)
13. Affective Attitude 1	0.89	3.59 (0.99)
14. Affective Attitude 2	0.75	3.90 (0.88)
15. Affective Attitude 3	0.88	3.46 (1.02)
16. Cues 1	0.72	3.29 (1.23)
17. Cues 2	0.82	3.28 (1.20)
18. Cues 3	0.63	3.72 (0.99)
19. Barriers 1	0.67	2.70 (1.15)
20. Barriers 2	0.80	2.52 (1.18)
21. Barriers 3	0.79	2.47 (1.23)
22. Barriers 4	0.75	2.42 (1.09)
23. Facilitators 1	0.66	4.15 (0.86)
24. Facilitators 2	0.64	3.86 (1.02)
25. Facilitators 3	0.78	4.06 (0.98)
26. Response S.E. 1	0.88	3.48 (1.05)
27. Response S.E. 2	0.86	3.39 (1.07)
28. Response S.E. 3	0.76	3.46 (1.10)
29. Task S.E. 1	0.69	3.93 (0.90)
30. Task S.E. 2	0.70	3.93 (0.84)
31. Task S.E. 3	0.78	3.69 (1.06)
34. Task S.E. 4	0.79	4.08 (0.91)
35. Task S.E. 5	0.86	4.07 (0.88)
36. Task S.E. 6	0.77	4.15 (0.92)
37. Self-administered Massage Behavior		60.10 (72.28)

Note: S.E denotes self-efficacy.

### 4.3 Direct effects

Supporting H_1_, intention (β = 0.21, *p* < .014) predicted S-KM behavior. Intention in turn was predicted by cues (H_2_: β = 0.29, *p* < 0.001), severity (H_3_: β = 0.27, *p* < 0.001), affective attitudes (H_5_: β = 0.14, *p* *=* *0*.011), task self-efficacy (H_7_: β = 0.29, *p* < 0.001), facilitators (H_9_: β = 0.22, *p* < 0.001), but not susceptibility (H_4_: β = 0.01, *p* = 0.827), instrumental attitudes (H_6_: β = 0.10, *p =* .058), response self-efficacy (H_8_: β = 0.09, *p =* .093), or barriers (H_10_: β = −0.08, *p* *=* *0*.160).

### 4.4 Indirect effects

When accounting for indirect effects, intention was found to mediate between i) cues and behavior (H_11_: β = 0.06, 95% CI 0.025, 0.129), ii) severity and behavior (H_12_: β = 0.06, 95% CI 0.014, 0.127). Model determinants were found to mediate between age and behavior (H_13_: β = −0.16, 95% CI −0.224, −0.093).

### 4.5 Antecedents

With regards to antecedents, gender and income did not show any predictive effects (*p*> .05). Age demonstrated the greatest number of predicted effects including instrumental attitudes (H_E_: β = −0.16, *p* = 0.014), severity (H_E_: β = −0.24, *p* < 0.001), barriers (H_E_: β = −0.20, *p* < 0.001), response self-efficacy (H_E_: β = −0.19, *p* *=* *0*.002), and affective attitudes (H_E_: β = −0.15, *p* *=* *0*.019). Lifetime MT also predicted several determinants that included task self-efficacy (H_E_: β = −0.19, *p* < 0.003), response self-efficacy (H_E_: β = 0.17, *p* = 0.006), affective attitudes (H_E_: β = −0.15, *p* *=* *0*.017), severity (H_E_: β = 0.26, *p* < 0.001), susceptibility (H_E_: β = 0.15, *p* = 0.010), facilitators (H_E_: β = −0.27, *p* < 0.001), and barriers (H_E_: β = 0.31, *p* < 0.001). Perceived health predicted severity (H_E_: β = −0.31, *p* < 0.001), susceptibility (H_E_: β =- 0.33, *p* < 0.001). barriers (H_E_: β = −0.17, *p* = 0.006).

### 4.6 Qualitative analysis on performing knee massage

A total of 197 participants responded to the qualitative question about performing massage for their knee. Most common strategies and techniques were: 1) Rubbing with the palm, thumb, and fingers around kneecap and stroking; ID: P43 “Circular motion mainly on lateral aspect of knee but completely circle at joint level.”, ID: P215 “Gliding, tapping, …more rhythmically massage”, ID: P243 “I rub the knee area in circular motions with my dominant hand, using my thumb to apply pressure to various spots surrounding the entire kneecap.”, ID:P229 “Without thought, I find myself stroking, soothing my knees in a rhythmic circular motion until the knees feel warm and pain seems less.”2) Deep tissue massage and kneading with pressure point therapy; ID:P187“I will also put pressure on a certain spot right above the kneecap and this helps the pain when it gets its worst.”, ID:P77“light pressure along the knee cab to help move the inflammation”, ID:P206“Sit upright and slowly put pressure on both sides of the knee for 2 to 3 min. I do these 10 to 15 times on each knee followed by wet heat compresses.”, ID: P263“Deep massage, high pressure and then rolling the fingers”, 3) Using electric massagers; ID:P 24 “I basically gently rub the area where it is most painful. I have used a handheld electric massager too.”, ID: P167“…I use an electric massage gun, I use a smaller vibrator to get right into the back of the knee”, ID: P88“Sometimes I rub around the area and sometimes I tap the area… I use an electric massager.”, ID:P 260“Start using coconut oil and hand massaging the entire knee especially the back and right side. Then I stretch it then have an electric massager that I hold on the areas that just the most. Then I will wrap the knee in a heating blanket for 30 min.”, 4) Applying massage techniques with ointment, cream, and oil; ID: P34 “Apply some Lidocaine ointment and massage in a roundabout circle on the whole knee very gently.”, ID: P94“I apply nerves and bone creams and gently massage the knee for 10–15 min most nights before going to bed.”, ID: P6 “I massage slowly with a muscular pain relieving cream.”, ID: P38 “Using Arnica Oil to massage into the knee joint”.

A small number of participants reported acquiring knowledge on how to massage knees from health care professionals; ID:P 37“Chiropractor showed me a few things. Including stretches to help with knee and hip, where to massage the tendons and the little lubricating spot above the knee. Including heat/cold, icing both my knees and lower back.”, ID:P 240 “I do what my therapist recommended, a series of movement stretches and massages”.

Comments were identified indicating massaging knee with automaticity; ID: P200“… Then I just manipulate my kneecap and surrounding area. On bad days my knees actually swell up and the pressure underneath feels like it could blow up my knee cap. I massage quite often even when not planned. It’s become a habit to help pain.”, ID: P229 ‘Without thought, I find myself stroking, soothing my knees in a rhythmic circular motion until the knees feel warm and pain seems less.’

## 5 Discussion

Self-administered massage is an effective self-management technique to manage symptoms in musculoskeletal conditions such as knee osteoarthritis. However, the inconsistent experimental designs such as the absence of a framework, theory or model to design intervention to promote S-KM behavior suggest that our understanding of the behavioral determinants associated to behavior change is still limited. The incorporation of health behavior theories cannot be ignored since the National Institutes of Health has urged researchers and practitioners to include conceptual models in their responses to proposals related to health promotion research (Brady et al., 2020). Hence, the purpose of this study was to address a significant literature gap highlighted by the disparity between what is recommended by NIH and what has been implemented in self-administered massage research. To achieve this, we augmented the Health Belief Model to supplement with constructs found in the Theory of Planned Behavior, namely, intention and attitudes to provide a conceptual model that can guide intervention development for S-KM behaviors.

The model posits that S-KM behavior is proximally predicted by intention. This prediction is supported by cues, perceived severity and susceptibility, attitudes (instrumental and affective), and both task and response self-efficacy, as well as barriers and facilitators. Overall, hypothesized effects from the model were largely supported by the structural equation model. Congruent with the hypothesis, intention was found to predict S-KM. This finding is aligned with literature where intention is recognized to be a proximal predictor across several self-management health behaviors (Duarte et al., 2022; Tak et al., 2023). Ajzen posited the positioning of intention to reflect the summation of reasons why an individual would perform a behavior, thus reflecting as the final motivational strength to perform behavior.

Cues were found to be one of the strongest predictors of intention, which also demonstrated an indirect effect to predict S-KM behavior. Cues function as prompts to signal initiation of behavior and the construct assessed mostly reflected temporal/associated times of performing massage or paired with temporal consistency. Future work that facilitates cues can prompt participants to perform knee massage after certain events in the day. Research has indicated that there are specific times (e.g. early morning) and activities that can exacerbate pain in knee OA (Murphy et al., 2015). Providing prompts or cues might be beneficial to encourage participants to perform knee massages in accordance with cue events (before starting physical activity) during the day.

The remaining model constructs comprised of two sub-constructs or components (Fig. 1), where interestingly one component often demonstrated to have predictive effects on intention, supporting most of our hypotheses. Specifically, perceived threats, reflected by perceived susceptibility and severity, revealed only severity to predict intention. This finding suggests that individuals with knee OA in this sample did not believe that there is likelihood or vulnerability for their arthritis to deteriorate over time, yet the outcome of the medical and/or social consequences of having arthritis is severe. This could be attributed to the long duration of living with knee OA among the participants (see Table 2), leading them to perceive their knee condition as stable since they have had it for a significant period of time. Future research may consider perceived susceptibility for primary prevention where the long-term progress of the knee OA is not predictable for individuals.

Similarly, affective attitudes, which reflects positive feeling about performing S-KM predicted intention. However, instrumental attitudes, which represent beliefs about the benefits of the behavior, did not predict the behavior execution. This holds true even though the scores for instrumental activities were high in this study (as shown in Table 2). Such a discrepancy between belief about the benefits of a behavior and actual performance has been documented in the literature for certain health behaviors, such as exercise. Although the significant health benefits of regular exercise are widely recognized, a large proportion of people fail to meet the recommended activity guidelines (King et al., 2019).

Additionally, task but not response self-efficacy was found to predict intention. These findings are interesting as participants believed that they were capable of performing S-KM but were not convinced that performing the behavior for 10-min per day would yield favorable health outcome specific to them. In future research, it may be crucial to view S-KM not as a standalone modality for intervention, but rather as a co-adjuvant practice that can be combined with therapist applied massage, exercise or conventional medicine.

The model found facilitators to be significant predictors and barriers to be non-significant predictors of intention, in line with the study hypothesis. While barriers are often a deterrent for several health-related behaviors (King et al., 2019), the flexibility of performing massage across environments and ability of quick behavioral enactment suggests barriers are not a concern for performing S-KM. However, facilitators that participants have time and energy are encouraging to support behavioral intervention.

Tests on antecedents revealed lifetime therapist applied experience, which signifies past experience was one of the strongest determinant. Receiving massage from trained professional likely revealed positive outcomes that might impact attitude towards performing S-KM (Munk et al., 2020). Future research can take into consideration cost effective interventions by short sessions of intervention that begins with performing massage on patients by a therapist and continues by instruction on techniques can facilitate task self-efficacy. Among all determinants, age was found to have a negative, dampening effect on determinants, suggesting that less strength of determinants among older adults. This pattern indicates that older adults with knee OA may have distinct intervention development needs that were not explored in this study.

Finally, it is worth addressing potential interpretations of non-significant pathways. Instrumental attitudes, perceived susceptibility, and response self-efficacy were found to correlate with intention; however, when tested across all model variables, these effects did not achieve statistical significance. Further investigation is warranted to determine why these determinants did not hold significance in the model. Taken together, these findings suggest that participants would benefit from education interventions that informs individuals on how to perform effective S-KM (task self-efficacy) with contextual cues to prompt behavior, which results in alleviating symptoms (affective attitudes). Facilitating these constructs would strengthen intention, and in turn increase likelihood of behavioral engagement that might result in improving symptoms.

The analysis of qualitative data indicated that the majority of participants utilized techniques resembling Swedish massage techniques. This may suggest that individuals acquire familiarity with these techniques through receiving massages from professional therapists as Swedish massage is widely practices in the US. Some participants reported using massage devices to massage their knees. This may indicate a potential shift towards the use of massage technology in S-KM future research to explore the effectiveness and safety of using massage devices.

### 5.1 Strengths and limitations

The study includes recognizable strengths in the scope of some limitations which should be acknowledged. Self-administered knee massage behavior was measured using self-reported questionnaire, and while objective measurements are preferred in health behavior research, there currently are no established instruments to assess S-KM behavior. The generalizability may have some limitations as the current sample was underrepresented in males and minorities. To our knowledge, the study is the most comprehensive theoretical investigation to identify determinants of self-administered massage behavior. Previous research has employed exploratory and general, traditional approaches, which supported and set the groundwork for the present investigation. The study provides novel intervention notes, supported by theoretical findings to design future interventions.

## 6 Conclusions

The S-KM literature for musculoskeletal conditions such as knee OA has made significant strides in understanding demographics, however, less advancements have made on the theoretical front, specifically for understanding behavioral determinants that would yield effective experimental designs. The present findings build on previous literature and contribute a comprehensive set of notes that warrant investigation for intervention. Specifically, cues, severity, task-self efficacy were found to be the strongest predictors of intention to perform self-administered massage, and hence, the facilitation of these constructs are recommended for future interventions. While these findings serve as some translational notes for generalizing to self-care, intervention investigations are warranted to test these techniques. Finally, the present findings can serve as a platform to advance theoretical investigations. Still, much work is needed to recognize constructs that were not specifically related to self-administered massage behavior but may impact behavioral adherence, such as age. Future intervention designs that are based on age groups could be more effective than a collective approach.

## Funding

This work did not receive funding.

## CRediT authorship contribution statement

**Donya Nemati:** Writing – original draft, Methodology, Investigation, Formal analysis, Data curation. **Navin Kaushal:** Writing – review & editing, Validation, Methodology. **NiCole Keith:** Writing – review & editing, Methodology. **Niki Munk:** Writing – review & editing, Supervision, Methodology.

## Declaration of competing interest

The authors declare that they have no known competing financial interests or personal relationships that could have appeared to influence the work reported in this paper.
